# Supplementary material for: Shape–Preserved CoFeNi–MOF/NF Exhibiting Superior Performance for Overall Water Splitting across Alkaline and Neutral Conditions
Source: Materials (Basel). 2024 May 7;17(10):2195. doi: 10.3390/ma17102195 (PMC11123414; doi:10.3390/ma17102195)
Supplement: Supplementary file 1 [file materials-17-02195-s001.zip › materials-2957407-supplementary.pdf]

materials

MDPI

Supplementary materials

Shape−Preserved CoFeNi−MOF/NF Exhibiting Superior Performance for Overall Water Splitting Across Alkaline and Neutral Conditions

Yu Liu, Panpan Li, Zegao Wang and Liangjuan Gao *

College of Materials Science and Engineering, Sichuan University, Chengdu 610065, China
* Correspondence: lgao87@scu.edu.cn

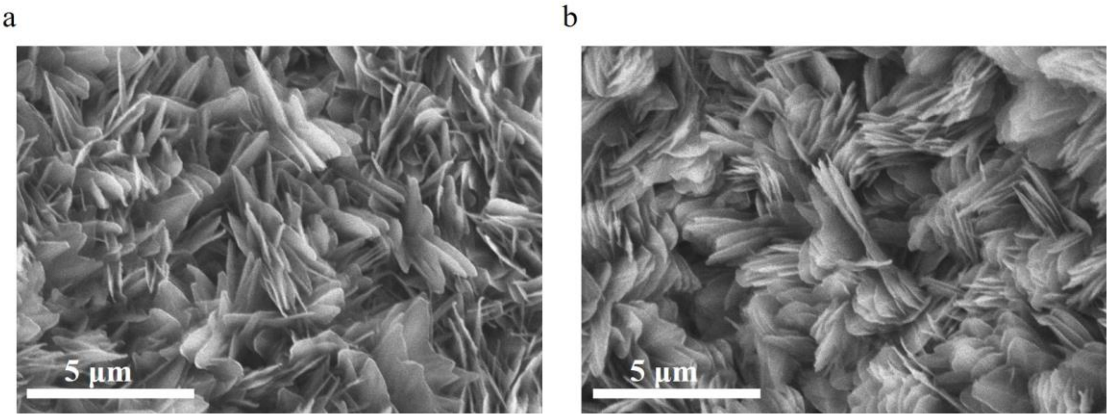

Figure S1. SEM images of (**a**) $Co_{0.45}Fe_{0.45}Ni_{0.9}$−LDH/NF and (**b**) $Co_{0.45}Fe_{0.45}Ni_{0.9}$−MOF/NF.

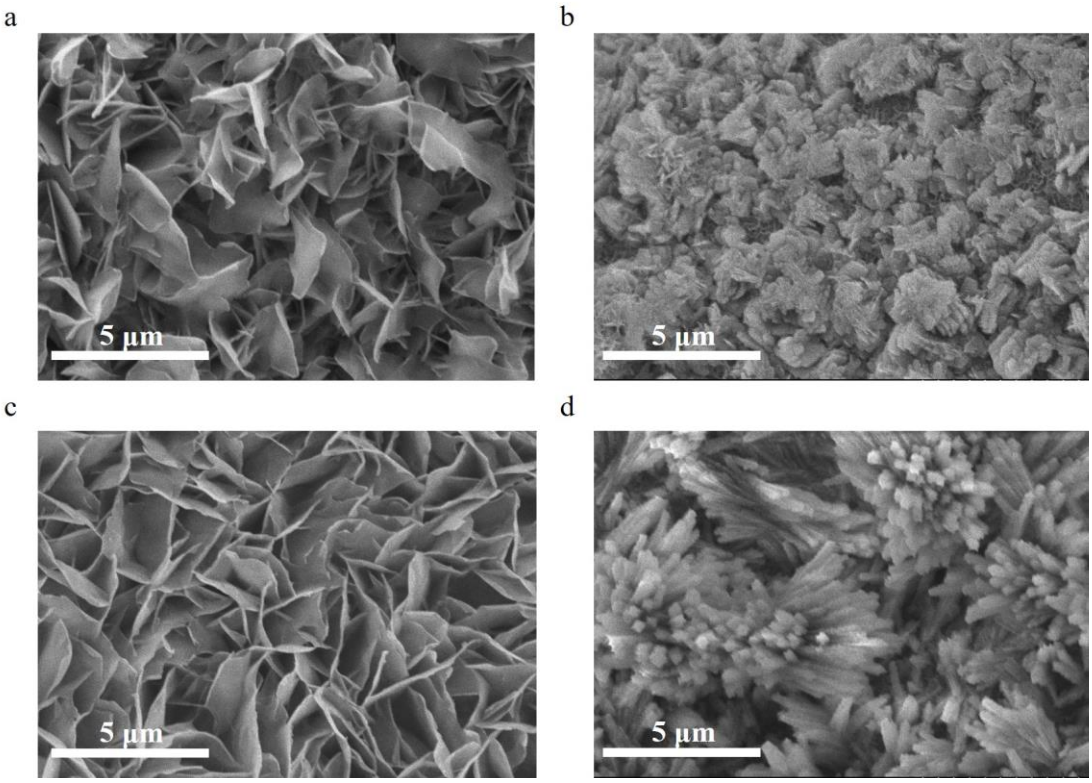

Figure S2. SEM images of (**a**) $Fe_{0.45}Ni_{0.9}$−MOF/NF; (**b**) $Co_{0.9}Fe_{0.45}Ni_{0.9}$−MOF/NF; (**c**) $Co_{0.45}Ni_{0.9}$−MOF/NF; and (**d**) $Co_{0.45}Fe_{0.9}Ni_{0.9}$−MOF/NF.

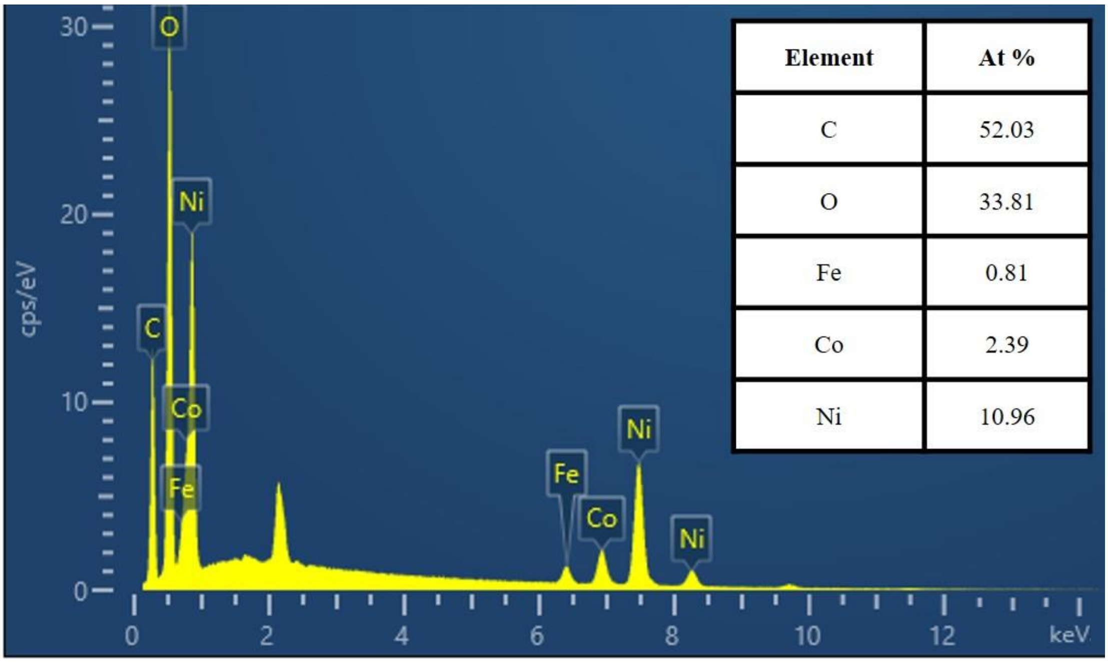

Element	At %
C	52.03
O	33.81
Fe	0.81
Co	2.39
Ni	10.96

Figure S3. EDS spectrum of $Co_{0.45}Fe_{0.45}Ni_{0.9}$−MOF/NF.

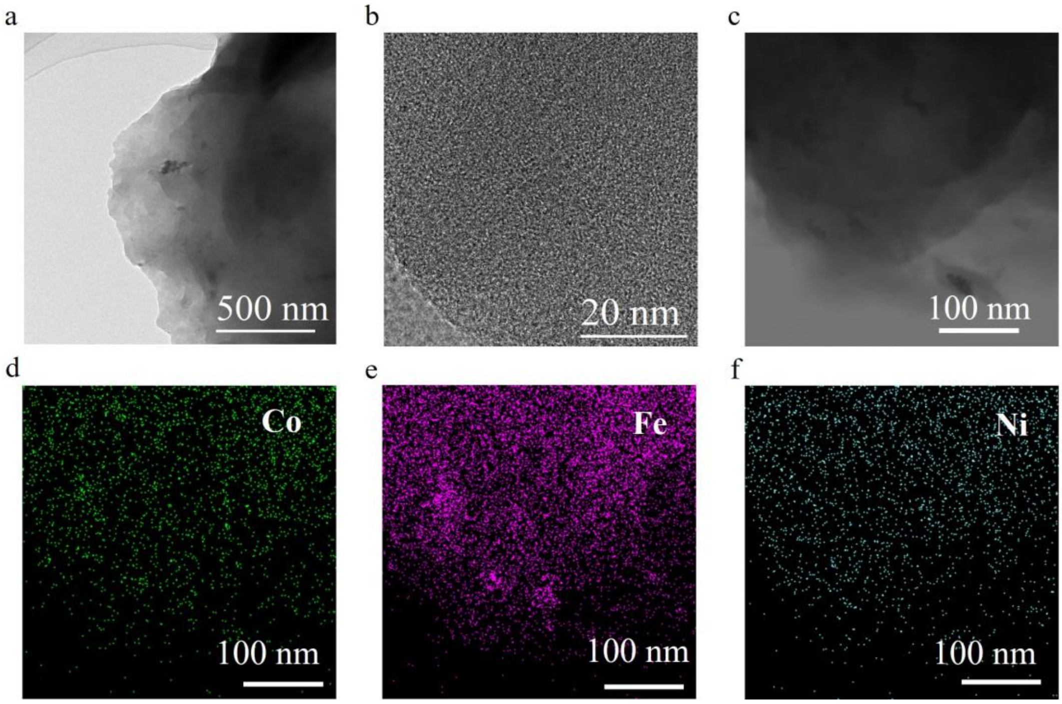

Figure S4. (**a**,**b**) HRTEM images and (**c**–**f**) EDS elemental mapping of $Co_{0.45}Fe_{0.45}Ni_{0.9}$–MOF/NF.

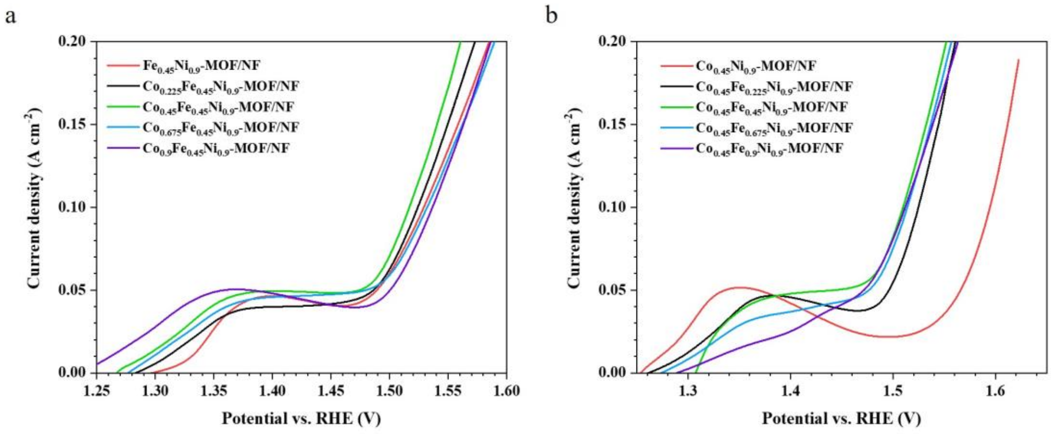

Figure S5. LSV curves of OER under 1 M KOH for the catalysts with different dosages of (**a**) Co and (**b**) Fe.

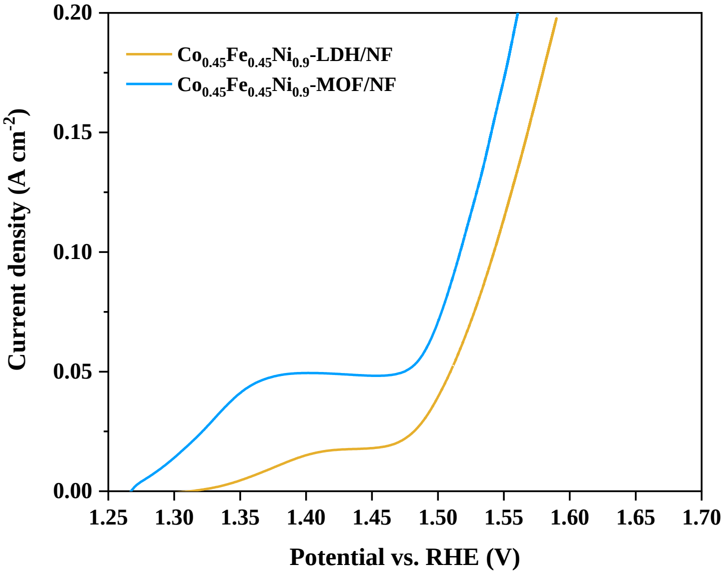

Figure S6. LSV curves of OER under 1 M KOH for $Co_{0.45}Fe_{0.45}Ni_{0.9}$–LDH/NF and $Co_{0.45}Fe_{0.45}Ni_{0.9}$–MOF/NF.

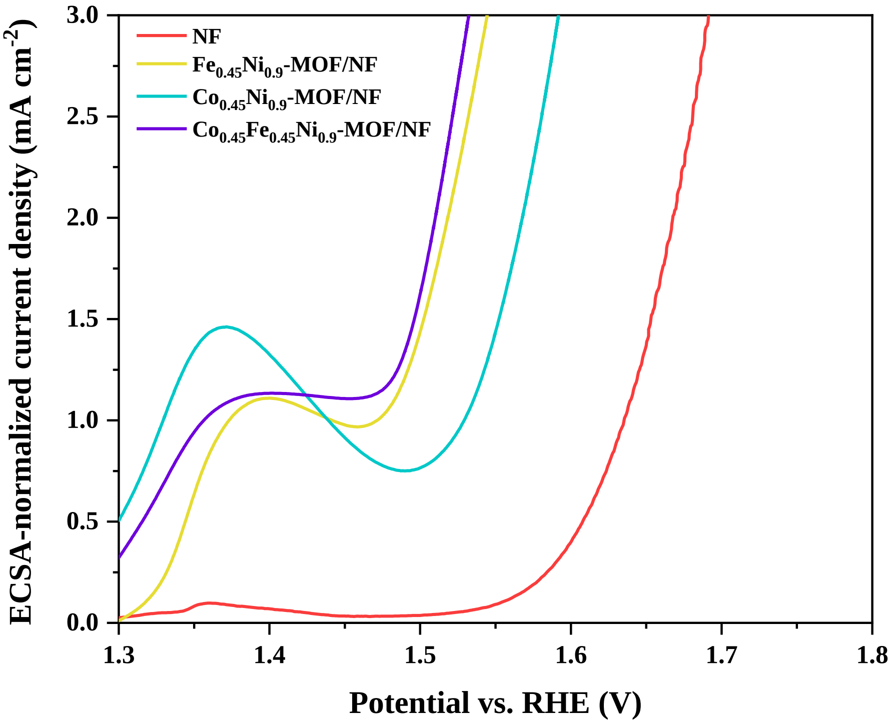

Figure S7. ECSA–normalized LSV curves of NF, $Fe_{0.45}Ni_{0.9}$–MOF/NF, $Co_{0.45}Ni_{0.9}$–MOF/NF, and $Co_{0.45}Fe_{0.45}Ni_{0.9}$–MOF/NF.

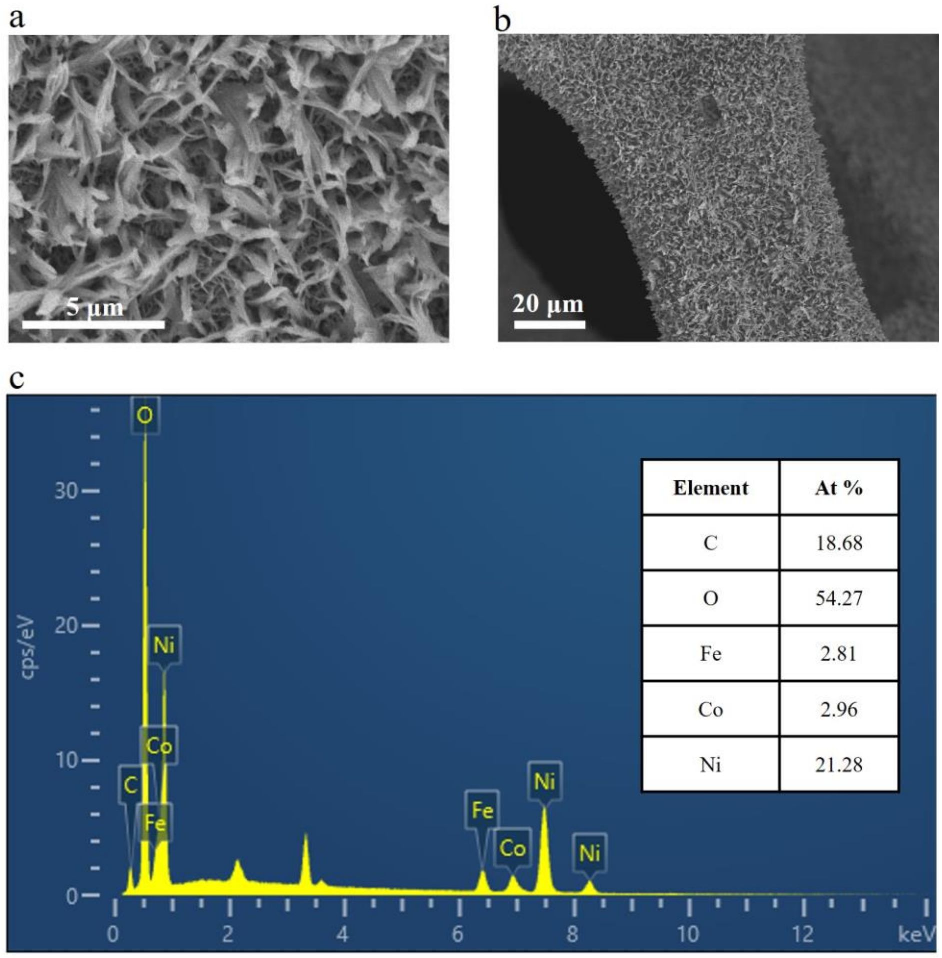

Figure S8. (**a**,**b**) SEM images of $Co_{0.45}Fe_{0.45}Ni_{0.9}$−MOF/NF after OER i−t test under 1 M KOH with different magnifications and (**c**) EDS spectrum of (**b**).

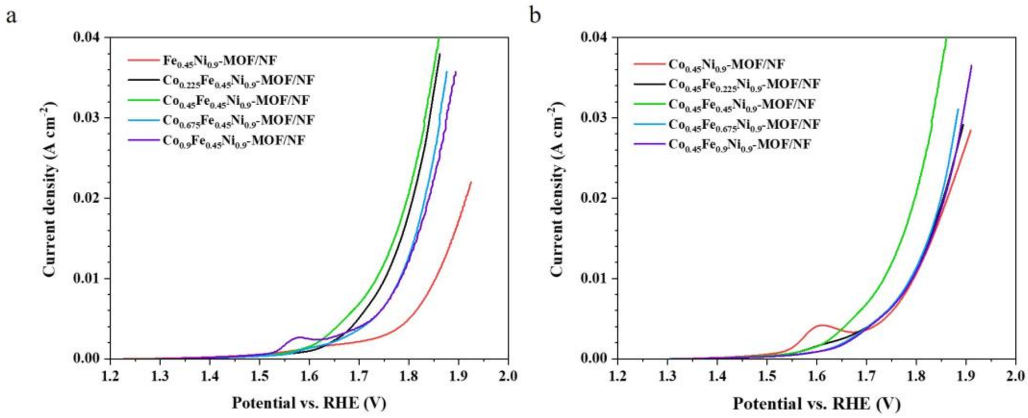

Figure S9. OER LSV curves under 1 M PBS for different dosages of (**a**) Co and (**b**) Fe.

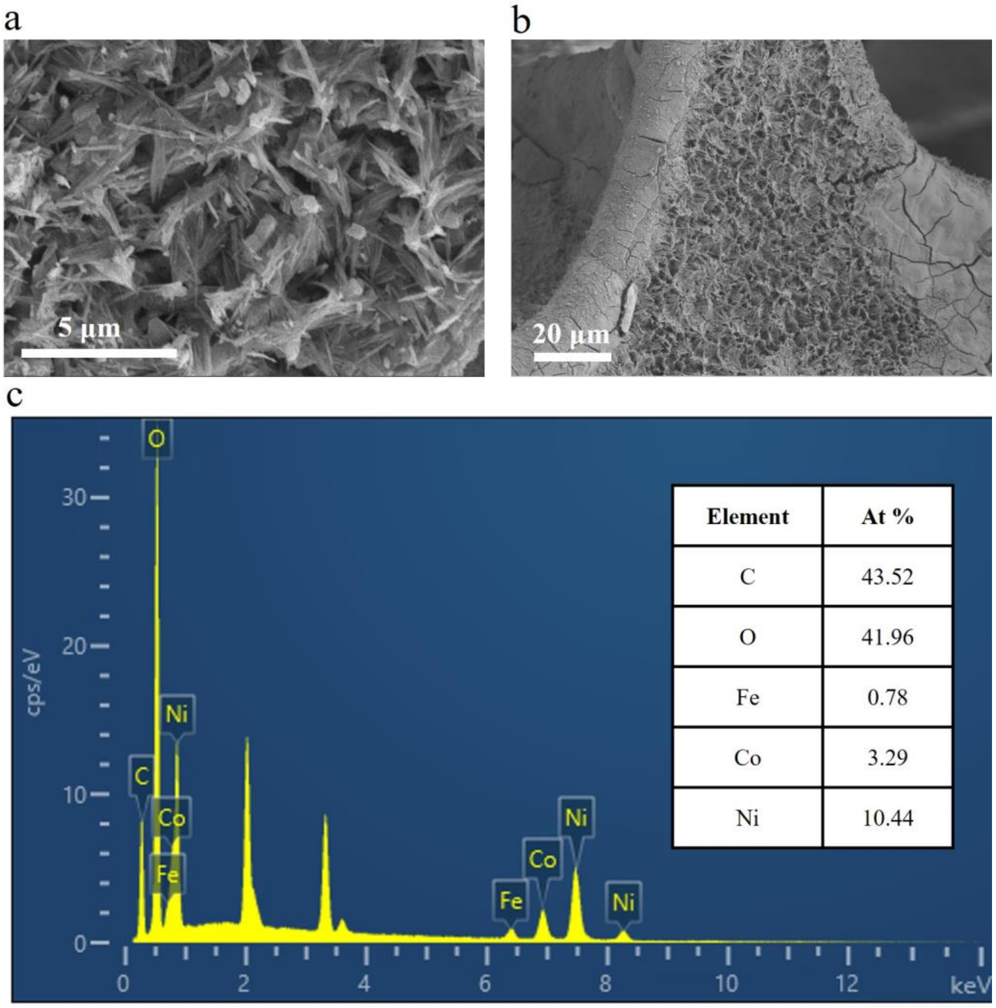

Figure S10. (**a**,**b**) SEM images of $Co_{0.45}Fe_{0.45}Ni_{0.9}$–MOF/NF after OER i–t test under 1 M PBS with different magnifications and (**c**) EDS spectrum of (**b**).

.

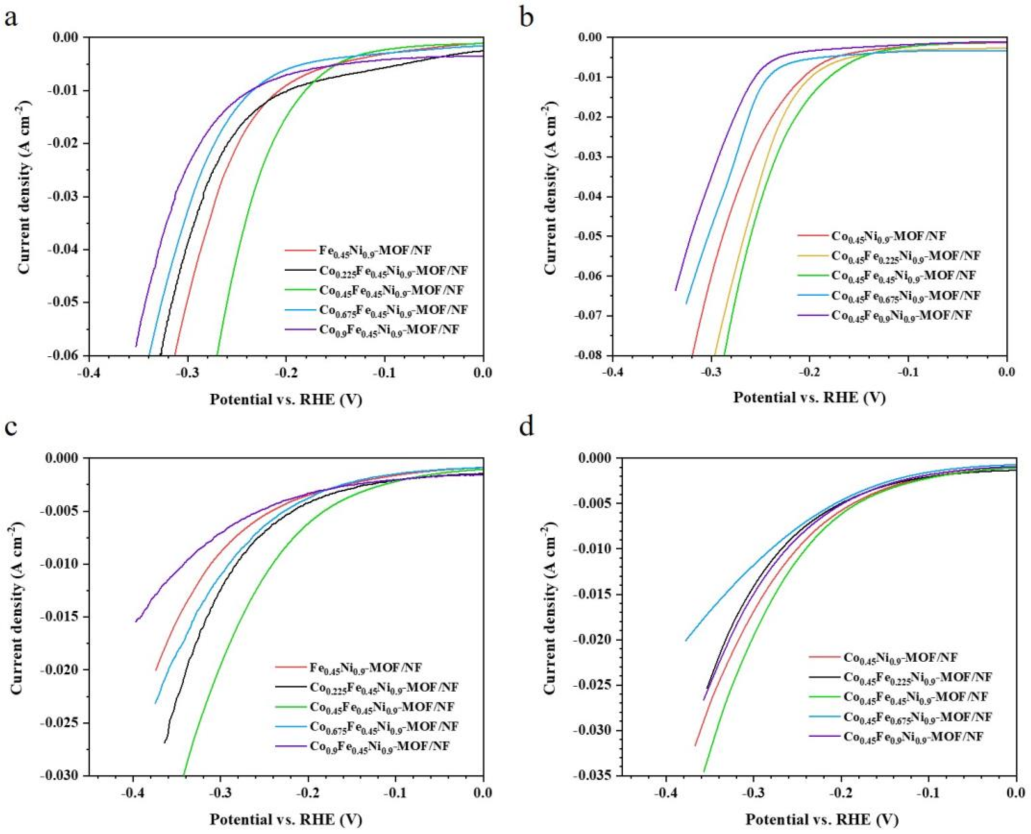

Figure S11. HER LSV curves under 1 M KOH with different dosages of (**a**) Co and (**b**) Fe. HER LSV curves at 1 M PBS with different dosages of (**c**) Co and (**d**) Fe.

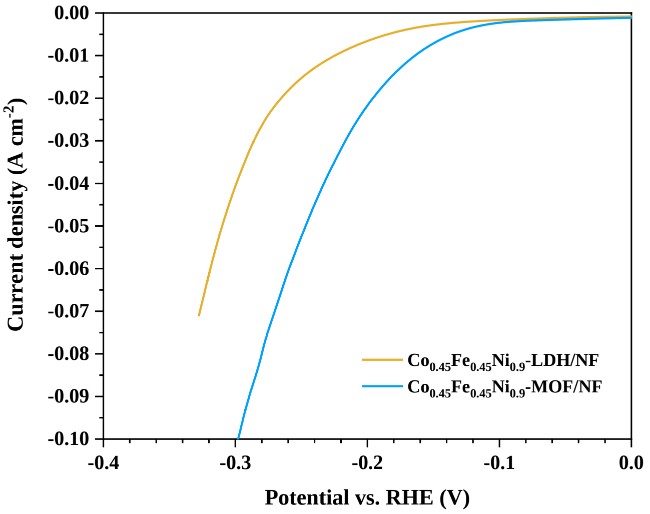

Figure S12. LSV curves of HER under 1 M KOH for $Co_{0.45}Fe_{0.45}Ni_{0.9}$–LDH/NF and $Co_{0.45}Fe_{0.45}Ni_{0.9}$–MOF/NF.

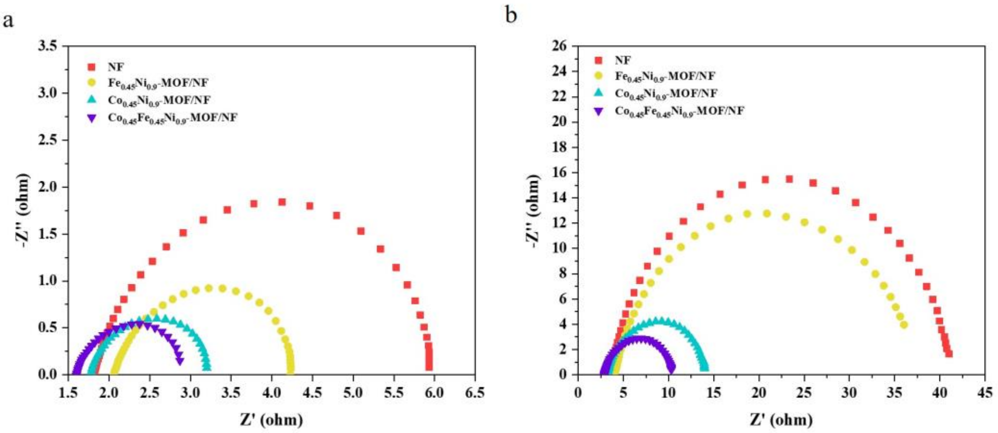

Figure S13. Nyquist plots measured at −0.3 V vs. RHE of NF, $Fe_{0.45}Ni_{0.9}$–MOF/NF, $Co_{0.45}Ni_{0.9}$–MOF/NF, and $Co_{0.45}Fe_{0.45}Ni_{0.9}$–MOF/NF under (**a**) 1 M KOH and (**b**) 1 M PBS, respectively.

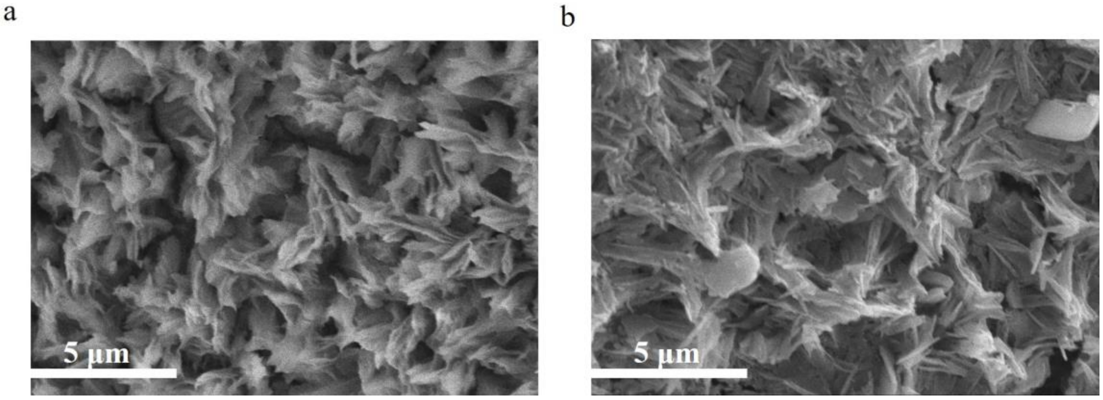

Figure S14. SEM images of $Co_{0.45}Fe_{0.45}Ni_{0.9}$–MOF/NF after the i–t test of HER under (**a**) 1 M KOH and (**b**) 1 M PBS, respectively.

Table S1. ICP–OES test of metallic elements in $Co_{0.45}Fe_{0.45}Ni_{0.9}$–MOF/NF.

Element	Mass concentration (mg/L)	Average mass concentration (mg/L)	Average atomic percentage (mol%)
Co	5.4	5.45	14.26
	5.5		
Fe	3.9	3.9	10.76
	3.9		
Ni	28.2	28.55	74.98
	28.9		

Table S2. Comparison of OER performance for $Co_{0.45}Fe_{0.45}Ni_{0.9}$–MOF/NF with other non–noble–metal–based catalysts under alkaline conditions.

Catalyst	j (mA cm^{-2})	η (mV)	Electrolyte	Ref.
$Co_{0.45}Fe_{0.45}Ni_{0.9}$–MOF/NF	50	244	1 M KOH	This work
	100	287		
$Ni_{10}Co$–BTC/KB	10	347	1 M KOH	[62]
Co−MOFs	50	348	1 M KOH	[57]

$FeNi_2S_4$	10	316	1 M KOH	[63]
NiCo–MOF/NF	50	270	1 M KOH	[64]
CVN/CC	10	263	1 M KOH	[65]
Ni(Fe)MOF/mKB14	50	291	1 M KOH	[66]
CoNi–CNT/NC–800	10	300	1 M KOH	[67]
Te–$Ni(OH)_2$/NF	10	270	1 M KOH	[68]
CoCu–MOF NBs	10	271	1 M KOH	[28]
Ni_2Mo_3N/NF	50	336	1 M KOH	[69]

Table S3. Comparison of OER performance for $Co_{0.45}Fe_{0.45}Ni_{0.9}$–MOF/NF with other non–noble–metal–based catalysts under near–neutral conditions.

Catalyst	*j* (mA cm^{-2})	η (mV)	Electrolyte	Ref.
$Co_{0.45}Fe_{0.45}Ni_{0.9}$–MOF/NF	10	505	1 M PBS	This work
Co–P_i/ITO	1	410	0.1 M KP_i	[70]
2D–Co–MOF	2	548	0.1 M SPB	[71]
Mn_5O_8 NP/FTO	5	580	0.3 M NaP_i	[72]
a–Co_2P	10	592	0.1 M PBS	[73]
Sub–10 MnO NC/FTO	5	530	0.3 M NaP_i	[74]
MnCat/ITO	1	590	0.1 M KP_i	[75]
$Co_{0.7}Fe_{0.3}P$/CNT	10	500	1 M PBS	[76]
Co–Ci–2D	1	480	0.1 M PBS	[77]
$Mn_3(PO_4)_2·3H_2O$/FTO	0.316	680	0.5 M NaP_i	[78]
Ni–Fe–Mg	10	514	0.5 M $KHCO_3$	[79]

Table S4. Comparison of HER performance for $Co_{0.45}Fe_{0.45}Ni_{0.9}$–MOF/NF with other non–noble–metal–based catalysts under alkaline conditions.

Catalyst	*j* (mA cm^{-2})	η (mV)	Electrolyte	Ref.
$Co_{0.45}Fe_{0.45}Ni_{0.9}$–MOF/NF	10	164	1 M KOH	This work
$FeNi_2S_4$	10	299	1 M KOH	[63]
CoNi−MOF−PCG	10	265	1 M KOH	[80]
MoS_2/MoO_3	10	210	1 M KOH	[81]
S−C−MoO_2/MoO_{3-x}	10	160	1 M KOH	[82]
N, S co–doped graphitic	10	230	0.1 M KOH	[83]
CuO@CQDs@CHNS	10	159	1 M KOH	[84]
CoS_2–600	10	270	1 M KOH	[85]
2D CoNi(1:1)–MOF	10	376	1 M KOH	[86]
Ni–doped CoP/Co_2P	10	161	1 M KOH	[87]
Co–BDC/MoS_2	10	248	1 M KOH	[88]

Table S5. Comparison of HER performance for $Co_{0.45}Fe_{0.45}Ni_{0.9}$–MOF/NF with other non–noble–metal–based catalysts under near–neutral conditions.

Catalyst	*j* (mA cm^{-2})	η (mV)	Electrolyte	Ref.
$Co_{0.45}Fe_{0.45}Ni_{0.9}$–MOF/NF	10	241	1 M PBS	This work
Ni–Cr–Mo–Fe	10	297	1 M PBS	[89]
CoP/CC	2	65	1 M PBS	[90]
NiP_x/CC	10	230	0.1 M ABS	[91]
CoO–$CoSe_2$	10	337	0.5 M PBS	[92]
$Ni_{0.1}Co_{0.9}P$	10	125	1 M PBS	[93]
SiO_2/PPy NTs–CFs	10	183	1 M PBS	[94]

FePN/CNT−200	10	158	1 M PBS	[95]
Ni−Co−Fe−P/NF	10	201	1 M PBS	[96]
CoFe@NC	10	340	1 M PBS	[97]
NiCo−MOF/NF	10	252	0.5 M Na_2SO_4	[18]

Table S6. Comparison of overall water splitting performance for $Co_{0.45}Fe_{0.45}Ni_{0.9}$−MOF/NF with other non−noble−metal−based catalysts under alkaline conditions.

**Catalyst**	***j*** **(mA cm⁻²)**	**Cell voltage (V)**	**Electrolyte**	**Ref.**
$Co_{0.45}Fe_{0.45}Ni_{0.9}$−MOF/NF	10	1.59	1 M KOH	This work
$FeNi_2S_4$	10	1.63	1 M KOH	[63]
CVN/CC	10	1.64	1 M KOH	[65]
CuO@CQDs@CHNS	10	1.83	1 M KOH	[84]
$Ni_3Fe_{0.9}Cr_{0.1}$/CACC	10	1.59	1 M KOH	[98]
Co−CeO_2@CNF/ Ni_2Fe@CNF	10	1.59	1 M KOH	[99]
NiCo−LDH−OH	10	1.89	1 M KOH	[100]
FeVO(OH)/$Ni(OH)_2$	20	1.65	1 M KOH	[101]
BP@MOF	10	1.63	1 M KOH	[102]
CoFe10%−P/NF	10	1.61	1 M KOH	[103]
$FeMn_6Ce_{0.5}$−MOF−74/NF	10	1.65	1 M KOH	[104]
